# Association study and expression analysis of olfactomedin like 3 gene related to meat quality, carcass characteristics, retail meat cut, and fatty acid composition in sheep

**DOI:** 10.5713/ab.21.0406

**Published:** 2022-05-02

**Authors:** Kasita Listyarini, Cece Sumantri, Sri Rahayu, Muhammad Jasim Uddin, Asep Gunawan

**Affiliations:** 1 Graduate School of Animal Production and Technology, Faculty of Animal Science, IPB University, Bogor, 16680, Indonesia; 2 Department of Animal Production and Technology, Faculty of Animal Science, IPB University, Bogor, 16680, Indonesia; 3 School of Veterinary Medicine, Murdoch University, Murdoch, WA 6150, Australia

**Keywords:** Carcass Characteristics, Fatty Acid Composition, Meat Cut, Meat Quality, *OLFML3*, Retail Sheep

## Abstract

**Objective:**

The objective of this study was to identify polymorphism in olfactomedin like 3 (*OLFML3*) gene, and association analysis with meat quality, carcass characteristics, retail meat cut, and fatty acid composition in sheep, and expression quantification of *OLFML3* gene in phenotypically divergent sheep.

**Methods:**

A total of 328 rams at the age of 10 to 12 months with an average body weight of 26.13 kg were used. A novel polymorphism was identified using high-throughput sequencing in sheep and genotyping of *OLFML3* polymorphism was performed using polymerase chain reaction-restriction fragment length polymorphism (PCR-RFLP). Among 328 rams, 100 rams representing various sheep genotypes were used for association study and proc general linear model was used to analyse association between genotypes and phenotypic traits. Quantitative real-time polymerase chain reaction (qRT-PCR) was used for the expression analysis of *OLFML3* mRNA in phenotypically divergent sheep population.

**Results:**

The findings revealed a novel polymorphism in the *OLFML3* gene (g.90317673 C>T). The *OLFML3* gene revealed three genotypes: CC, CT, and TT. The single nucleotide polymorphism (SNP) was found to be significantly (p<0.05) associated with meat quality traits such as tenderness and cooking loss; carcass characteristics such as carcass length; retail meat cut such as pelvic fat in leg, intramuscular fat in loin and tenderloin, muscle in flank and shank; fatty acids composition such as tridecanoic acid (C13:0), palmitoleic acid (C16:1), heptadecanoic acid (C17:0), ginkgolic acid (C17:1), linolenic acid (C18:3n3), arachidic acid (C20:0), eicosenoic acid (C20:1), arachidonic acid (C20:4n6), heneicosylic acid (C21:0), and nervonic acid (C24:1). The TT genotype was associated with higher level of meat quality, carcass characteristics, retail meat cut, and some fatty acids composition. However, the mRNA expression analysis was not different among genotypes.

**Conclusion:**

The *OLFML3* gene could be a potential putative candidate for selecting higher quality sheep meat, carcass characteristics, retail meat cuts, and fatty acid composition in sheep.

## INTRODUCTION

Sheep meat is one of the most important protein source in Indonesia beside poultry and beef. Indonesia is a tropical country which has a suitable climate for the growth and development of sheep. The adaptability of sheep in the local climatic condition makes them an important livestock in nation’s small scale farming system. Varities of Indonesian sheep breeds are popular in the country and notable breeds include JFT (Javanese fat tailed), JTT (Javanese thin tailed), GCS (Garut composite sheep), BCS (Barbados cross sheep), CAS (Compass agrinak sheep), GS (Garut sheep), and JS (Jonggol sheep). JFT sheep are white and hornless, and is a predominant breed in East Java [1], whereas JTT are small and generally white breed predominant in West Java [1,2]. GCS are crossbred with indigenous GS (50%), St. Croix sheep from the Virgin Islands (25%), and Moulton Charolais sheep from France (25%). The GCS has individual calf weights that are higher than Garut sheep, ranging from birth weight to 12 months of age [3]. BCS are crossbred with local Sumatera (50%) and Barbados blackbelly (50%) [4], whereas CAS are crossbred with local Sumatera (50%), St. Croix sheep (25%), and Barbados blackbelly (25%) [5]. GS are popular fighting sheep, distinguished from other thin-tailed sheep by their bigger stature and convex facial profile [2]. JS are crossbred with thin tailed sheep (50%) and GS (50%) and have no or less fat, white and black stripes fleece, and rams have horns while ewes don’t have horns [6]. These sheep are playing a povital role not only as protein source but also for the poverty alleviation in Indonesia, a developing country with a large polulation. Therefore, it is utmost important to understand their genetic makeup and meat quality.

Meat quality and carcass characteristics determine the quality of meat and the value of animal for consumer, producer, and meat industry [7]. Because of their economic value, these are the key triats in sheep breeding programs. Besides productivity traits, carcass and meat quality traits must be consistently enhanced or managed at optimal level to ensure the lamb industry’s long-term competitiveness [8]. Meat quality and carcass characteristics are complex traits that include meat flavor, tenderness, colour, juciness, mineral content, muscle oxidative capacity, pre-slaughter weight, dressing out percentage, cold carcass weight, hot carcass weight, lean weight, bone weight, retail-cut, fat weight, and fatty acids (FA) composition traits. Among them flavor is identidied as the most important trait [9]. Note, the flavor is affected by FA composition. The composition of FA determines the firmness of adipose tissue and the oxidative stability of muscle [10]. Nevertheless, FA composition and adipose tissues are essential for the nutritional value of meat [10], as well as they affects meat quality, and influence flavor, juiciness, and tenderness [11]. Thus, improving meat quality, carcass characteristics, retail meat cut, and FA composition of meat are pivotal for sustainable sheep production. Genetic methods and breeding are suggested as one of the most feasible methods for improving these traits. Van der Steen et al [12] reported that breeding programs were most efficiently performed using molecular genetics of candidate genes approach through variant analysis. Meat quality traits are found to be of moderately heritabile, range between 0.15 and 0.46 [13] which indicates that selection will be more effective to improve genetic merit for these traits. Therefore, genetic factors or markers controlling meat quality, carcass characteristics, retail meat cut, and FA composition could be implemented in breeding programmes.

Olfactomedin like 3 ( *OLFML3*) gene (Gene ID: 101121985) encodes an extracellular matrix glycoprotein which takes part in different physiological processes such as embryo development, and facilitates protein-protein interactions, cell adhesion, and intercellular interactions. *OLFML3* is reported to influence meat quality traits especially meat tenderness in cattle [14]. *OLFML3* gene is involved in myogenesis including muscle tissue development during embryonic development, and maintenance of mature skeletal musculature and tissue homeostasis [15]. *OLFML3* gene is shown to exhibit differential expression in skeletal muscle at prenatal and postnatal developmental stages in pigs [15]. The *OLFML3* gene is located in Chromosome 1 (Primary_assembly 1: 96,121,872–96,124,453, ENSEMBL) in sheep, and the region is detected to harbour several quantitative trait loci (QTL) influencing meat quality traits such as backfat thickness at 3rd lumber [16], muscle (Longissimus dorsi) depth at 3rd lumber [16], average daily gain and body mass [17,18], bone mass and dressed carcass bone percentage [19] in sheep. These data indicated that *OLFML3* could be a potential positional candidate gene for meat quality traits in sheep. Notably, a number of candidate genes have been identified for meat quality, carcass characteristics, retail meat cut, and FA composition in sheep including calpastatin (*CAST*) [20] for carcass characteristics; cytochrome P450, family 2, subfamily A, polypeptide 6 (*CYP2A6*) and kinesin family member 12 (*KIF12*) genes for flavor and odour [21], and THO complex 5 (*THOC5*) gene [22] for FA composition. Despite promising QTL and functional data, association and expression study of *OLFML3* gene related to meat quality, carcass characteristics, retail meat cut, and FA composition has not been done particularly in sheep. Therefore, the aim of this study was to analyse polymorphism, association and expression study of *OLFML3* gene with regards to meat quality, carcass characteristics, retail meat cut, and FA composition in sheep.

## MATERIALS AND METHODS

### Animals and phenotypes

All procedures involving animals were approved by the Animal Ethics Commission of the Institut Pertanian Bogor (IPB) University (approval no.117-2018 IPB). A total of 328 rams from seven different populations were used for identification of gene polymorphism in this study including JFT (n = 20), JTT (n = 76), GCS (n = 45), BCS (n = 36), CAS (n = 35), GS (n = 20), and JS (n = 96). Data on meat quality and carcass characteristics were obtained from rams with body weight between 25 to 30 kg, and aged between 10 to 12 months. The rams were reared under the same management systems and were fed the same fattening feed (grass by 10% of body weight and concentrate GT-03) and water *ad libitum*. A total of 100 rams representing various sheep genotypes including JFT (n = 15), JTT (n = 20), GCS (n = 10), BCS (n = 10), CAS (n = 10), GS (n = 20), and JS (n = 15) were slaughtered in a commercial abattoir PT Pramana Pangan Utama (PPU) Slaughter House and were used for association study. Slaughtering was carried out according to guidelines of the Indonesian performance test. All carcasses were cuts into two parts (right and left) after storing at −20°C temperature for 24 hours. The carcass on the right were cuts into seven pieces of commercial cuts (leg, loin, rack, neck, shoulder, breast-fore shank, and flank), then it was divided into meat, bone, subcutaneous and intramuscular fat. All samples for the meat quality, carcass characteristics, FA composition analysis, and deoxyribonucleic acid (DNA) extraction were performed on ice and stored at −20°C.

### Meat quality and carcass characteristics traits analysis

Meat quality traits were analysed including pH (power of hydrogen) value, tenderness, cooking loss, and water holding capacity (WHC) [20]. The pH value was measured with a pH meter after carcass being stored for 24 hours postmortem (final pH). Meat tenderness was measured using warner bratzler shear force (WBSF) [20]. Cooking loss was measured by deducting the initial weight of the sample meat after being cooked in a waterbath at a temperature of 80°C for 1 hour [20]. The WHC was measured by finding the amount of water that comes out (mgH_2_O). WHC is the percentage of weight lost of 5 g meat samples after being pressurized 2,250 g for 5 minutes [23].

Carcass characteristics were measured including body weight, hot carcass weight, cold carcass weight, dressing percentage, and carcass length [20]. Live body weight was calculated before the rams is slaughtered. Hot carcass weight was measured after all non-carcass components (head, skin, heart, liver, lungs, trachea, spleen, and ducts digestion) were removed [20]. Cold carcass weight was measured after the carcass was stored at −20°C for 24 hours. Dressing percentage was calculated based on empty body weight [20]. Empty body weight is the live body weight minus the contents of the digestive tract. Carcass length was measured from point of the shoulder to the distal end of the tarsus [20].

### Fatty acid composition analysis

Longissimus dorsi muscle samples were analyzed for FA composition. Each sample of fat and FA composition was determined using the Association of Official Analytical Chemists (AOAC) 991.36 and AOAC 969.333 [22] extraction methods. The FA composition was measured using gas chromatography (GC), which usually requires fat extraction, methylation, conversion of the FA into methyl esters, and finally determination by GC. Saturated, monounsaturated, and polyunsaturated fatty acid levels were measured [22].

### DNA extraction and polymerase chain reaction-restriction fragment length polymorphism amplification

Genomic DNA was extracted from longissimus dorsi muscle samples using the Geneaid gSYNC DNA Extraction Kit (Catalogue Number: GS050/100/300) based on the manufacturing protocol. A novel polymorphism (g.90317673 C>T) was identied in *OLFML3* gene (Gene ID: 101121985) using high-throughput sequeicng in sheep. Gene-specific primers were designed using MEGA 6.0, and Primer Stat was used to determine the suitability of polymerase chain reaction (PCR) tests (Table 1). The PCR was carried out in a 15 μL reaction that contained 1 μL of genomic DNA, 0.2 μL of forward and reverse primers, 7.5 μL of MyTaq Red Mix, and 6.1 μL of ddH_2_O. The following thermocycling profile was used to amplify *OLFML3* gene (Gene ID: 101121985) fragments using the ESCO GeneAmp PCR system: initial denaturation at 94°C for 1 min, followed by 35 cycles at 94°C for 10 s, 59°C for 15 s, 72°C for 15 s, and final extention at 72°C for 1 min. The DNA amplicon was viewed by 1.5 percent electrophoresis gel. PCR-restriction fragment length polymorphism (PCR-RFLP) was used to perform single nucleotide polymorphism (SNP) genotyping validation. PCR-RFLP approach involves digesting PCR amplicons with appropriate restriction enzymes to produce distinct polymorphic fragments used as species identification markers [24]. Five μL of DNA amplification product was digested in 0.7 μL buffer, 1 μL of ddH_2_O, and 0.3 μL of Msp*I* restriction enzymes (Thermo Scientific, Waltham, MA, USA) in a final volume of 7 μL, followed by an incubation at 37°C for 4 hours. The digested products were visualized using a 2 percent agarose gel. The genotypes of *OLFML3* gene were determined based on the length of the bands. The genotypes of *OLFML3* gene were: 195 and 303 bp for CC genotype; 195, 303, and 498 bp for CT genotype; and 498 bp for TT genotype.

### Expression analysis in sheep with divergent meat quality, carcass characteristics, retail meat cut, and fatty acid composition

Ribonucleic acid (RNA) was extracted from nine rams’ liver tissues using RNeasy Mini Kit (Qiagen, Venlo, Netherland) based on genotype results with different meat quality, carcass characteristics, retail meat cut, and FA composition. RNA was extracted from liver tissue because fat is metabolized in the liver and the *OLFML3* is abundantly expressed in liver [15]. Three groups, each consisting of three rams were formed based on CC, CT, and TT genotype. The procGLM test in statistical software (SAS) was used to measure the significant difference between the three groups. Complementary DNA (cDNA) was synthesized using a First Strand cDNA Kit (Thermo Scientific, USA) for the qRT-PCR analysis. Gene (XM_004002351.5) specific primers for the qRT-PCR analysis were designed in molecular evolutionary genetics analysis (MEGA) 6.0 (Table 1). Each cDNA sample and no-template control were examined in a 96-well microtiter plate in each run. All samples were tested twice, and the target gene quantification was normalized using geometric mean of two housekeeping genes (glyceraldehyde-3-phosphate dehydrogenase and *β-Actin*). AG qTower Analytic Jena program was conducted to quantify the cDNA [21].

### Data analysis

After obtaining genotypes data using the PCR-RFLP method, the allele frequency, genotype frequencies, and Hardy-Weinberg equilibrium (HWE) value were calculated according to Nei and Kumar [25]. Association of *OLFML3* gene with phenotypes was computed using SAS ver 9.2. PROC general linear model (GLM) using a fixed effect model (analysis of variance) was used to analyze the effects of genotype.


$$Y_{ijk}=\mu +{\text{genotype}}_{i}+{\text{breed}}_{j}+e_{ijk}$$

Where: *Y_ijk_* = the meat quality, carcass characteristics, retail meat cut, and FA composition; μ = the population mean; genotype*_i_* = the fixed effect of i-th genotype; breed*_j_* = the fixed effect of j-th breed; *e_ijk_* = the residual error.

For mRNA expression analysis, the delta Ct method, de scribed previously by Silver et al [26] was used for calculating the difference between target gene and the reference gene (ΔCt = Ct_target_ − Ct_housekeeping genes_). The expression of the gene was measured as fold change determined from ΔCt values. The phenotype differences for both high and low quality of meat, carcass characteristics, retail meat cut, and FA composition of *OLFML3* gene expressions were compared using *t*-tests. The p<0.05 values were considered to show significant differences.

## RESULTS

### Polymorphism of *OLFML3* gene

**Figure 1:**
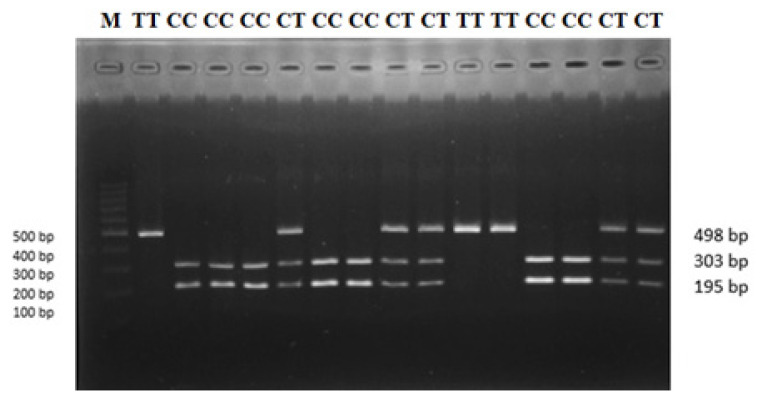
PCR-RFLP genotyping result for *OLFML3* gene revealed with MspI restriction enzymes. M, 100 bp ladder size standard; CC, CT, and TT are genotypes. PCR-RFLP, polymerase chain reaction-restriction fragment length polymorphism; *OLFML3*, olfactomedin like 3.

A SNP was identified in *OLFML3* gene at position g.90317673 C>T. Three genotypes CC, CT, and TT were found for SNP g.90317673 C>T in sheep populations used in this study (Figure 1). The SNP genotypes were: 195 and 303 bp for CC genotype; 195, 303, and 498 bp for CT genotype; 498 bp for TT genotype. The TT genotype was relatively rare in our populations (JFT, JTT, BCS, CAS, GS, and JS), except in GCS. The genotypes were detected in HWE. The detailed genotype and allele frequencies are showed in Table 2.

### Association of *OLFML3* gene polymorphism with meat quality, carcass characteristics, retail meat cut, and fatty acid composition

The results of the association analysis showed that *OLFML3* (g.90317673 C>T) was significantly (p<0.05) associated with meat quality traits such as tenderness and cooking loss; carcass characteristics such as carcass length (Table 3); retail meat cut such as pelvic fat in leg, intramuscular fat in loin and tenderloin, muscle in flank and shank, (Table 4); and FA composition such as tridecanoic acid (C13:0), palmitoleic acid (C16:1), heptadecanoic acid (C17:0), ginkgolic acid (C17:1), linolenic acid (C18:3n3), arachidic acid (C20:0), eicosenoic acid (C20:1), arachidonic acid (C20:4n6), heneicosylic acid (C21:0), and nervonic acid (C24:1) (Table 5). The TT genotype was associated with some of the meat quality, carcass characteristics, retail meat cut, and FA composition traits, where animals bearing TT genotype showed higher values for the traits (detailed in Table 3, 4, 5).

### Expression analysis of *OLFML3* gene

**Figure 2:**
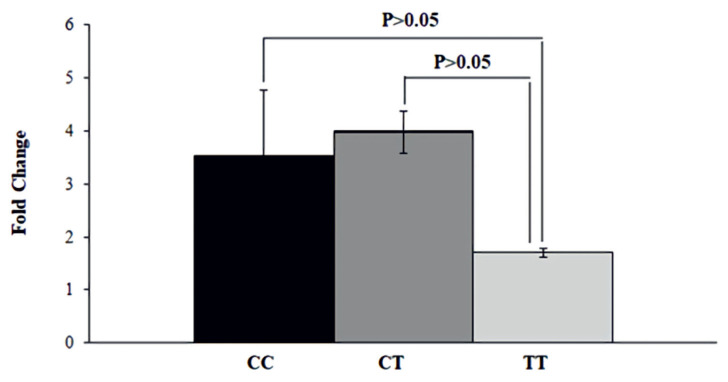
mRNA expression of *OLFML3* gene in animals with divergent genotypes for meat quality traits using qRT-PCR. The genotypes CC (black), CT (dark grey), and TT (light grey) were placed in X-axis and Y-axis represents fold change of gene expression quantified with qRT-PCR. The mRNA expression was not significantly different between CC, CT, and TT genotypes (p>0.05). *OLFML3*, olfactomedin like 3; qRT-PCR, quantitative real-time polymerase chain reaction.

Expression analysis of *OLFML3* gene showed that there was no significant differences in expression of transcript in rams with different genotypes. The messenger RNA (mRNA) expression was not significantly different between CC, CT, and TT genotypes (p>0.05) (Figure 2). Although it was not statistically significant (p>0.05), the mRNA expression showed a lower trend in TT genotype compared to CT and CC.

## DISCUSSION

For the polymorphism study, *OLFML3* gene fragment was amplified successfully using PCR-RFLP in all seven sheep populations. The *OLFML3* gene had three genotypes (CC, CT, and TT). The TT genotype was relatively rare in the populations (JFT, JTT, BCS, CAS, GS, and JS). This finding is similar to an association study which has used a different gene (diacylglycerol O-acyltransferase 1 [*DGAT1*]) in JFT, JTT, BCS, CAS, and GCS [27], however it is inconsistent with the previous study performed for a different gene (betaine-homocysteine S-methyltransferase [*BHMT*]) in JFT, JTT, BCS, CAS, and GCS [28]. Munyaneza et al [28] reported that TT genotypes are more frequent than CC and CT genotypes. The SNP of *OLFML3* gene was in HWE. *OLFML3* gene polymorphism is found to be significantly associated with meat quality traits including tenderness in this study. The CC genotype was associated with higher value of tenderness, while the TT genotype was associated with lower value of tenderness. A high value for meat tenderness indicates that the meat is tougher than that of a lower value meat. Sheep inheriting the TT genotype in *OLFML3* gene had more tender meat than those inheriting the CC and CT genotypes. Thus TT genotype is more desiable for good quality meat. Tenderness is a very important meat quality trait because variations in tenderness are suggested to affect the consumers’ decision to repurchase [29].

The SNP in *OLFML3* gene is found to be significantly associated with cooking loss in this study. The TT genotype is associated with higher level of cooking loss. These results are different than a previous study conducted with a different gene (*CAST*) in Javanese thin-tailed sheep [20]. A high cooking loss value indicates that high amount of water is lost from the meat during the boiling process. The percentage of cooking loss is positively correlated with the value of tenderness. Cooking loss was found to be increased with carcass weight in this study. These results are consistent with a previous study reported by Russo et al [30]. The *OLFML3* gene is significantly associated with carcass characteristics including carcass length. The CT and TT genotype were associated with higher level of carcass length. These findings are in consistent with a previous study performed with a different gene (*DGAT1*) in Indonesian sheep [27]. The leg has a higher weight of muscle, bone, and subcutaneous fat compared to other parts (shoulder, shank, breast, neck, rack, loin, flank, and tenderloin). On the other hand, the leg has a lower intramuscular fat than the shoulder. The percentage of intramuscular fat in loin and flank are lower compared to that of in shoulder. This finding is in agreement with a study performed with a different gene (*CAST*) in Javanese thin-tailed sheep [20]. However, the SNP in *OLFML3* gene was significantly associated with retail meat cut including pelvic fat in leg, intramuscular fat in loin and tenderloin, muscle in flank and shank. The TT genotype was associated with higher level of pelvic fat in leg, intramuscular fat in loin and tenderloin, muscle in flank and shank. It is well known that intramuscular fat (marbling) keeps meat moist, increases the palatability and positively affects the consumers’ choice. The higher muscle is always desirable which is found to be associated with TT genotypes. Therefore, TT genotype is desirable for better retail cut traits than other genotypes.

The polymorphism in *OLFML3* gene was significantly associated with FAs composition including palmitoleic acid (C16:1), heptadecanoic acid (C17:0), linolenic acid (C18:3n3), arachidic acid (C20:0), eicosenoic acid (C20:1), heneicosylic acid (C21:0), and nervonic acid (C24:1). These results are in agreement with a previous study performed with alpha 2-heremans Schmid glycoprotein (*AHSG*) gene in Indonesian sheep population [31]. Monounsaturated FA including palmitoleic acid (C16:1) and nervonic acid (C24:1) have beneficial effects on health. Palmitoleic acid (C16:1) has the potential to be used as a therapeutic agent for metabolic syndrome, insulin resistance, and diabetes, whereas nervonic acid (C24:1) plays critical roles in brain development and promotes nerve cells regeneration. The *OLFML3* gene was significantly associated with FAs composition including tridecanoic acid (C13:0) and ginkgolic acid (C17:1). These results are in consistent with a previous study that performed with a different gene (17beta 13-Hydroxysteroid dehydrogenases [*HSD17β13*]) in Indonesian sheep [32]. Note, some FAs were higher in animals with CC genotype, while some FAs were higher in animals with TT genotype. These findings postulate that the polymorphism (g.90317673 C>T) in *OLFML3* gene may contribute in meat quality, carcass characteristic, retail meat cut, and FA composition in sheep.

The expression study revealed that the mRNA expression of *OLFML3* gene was not significantly (p>0.05) differently between CC, CT, and TT genotypes. A previous study reported that the *OLFML3* gene was abundantly expressed in liver and pancreas tissues [15]. The *OLFML3* gene is reported to be down-regulated in Landrace and Tongcheng pigs [15]. Ying and Ji-Liang [33] have reported that olfactomedin (*OLF*) family gene *OLFML3* encodes a glycoprotein with 406 amino acid residues which is unique in the family. Because of the unique structure and differential expression, the *OLFML3* gene could have biological functions that are distinct from those of other members of the *OLF* family. This finding suggests that *OLFML3* gene may contribute to meat quality, carcass characteristic, retail meat cut, and FA composition in sheep.

## CONCLUSION

The *OLFML3* gene was polymorphic in different Indonesian sheep such as JFT, JTT, GCS, BCS, CAS, GS, and JS. The SNP g.90317673 C>T in *OLFML3* gene was significantly associated with tenderness, cooking loss, carcass length, pelvic fat in leg, intramuscular fat in loin and tenderloin, muscle in flank and shank, tridecanoic acid, palmitoleic acid, heptadecanoic acid, ginkgolic acid, linolenic acid, arachidic acid, eicosenoic acid, arachidonic acid, heneicosylic acid, and nervonic acid. The values of some favourable traits were higher in animals with TT genotype. Therefore, TT genotype is desirable for some favourable traits than other genotypes, but the rarity of the genotype should be noted. Finally, it could be postulated that the polymorphism in *OLFML3* gene could be a potential marker for meat quality, carcass characteristic, retail meat cut, and fatty acids composition in sheep.

## Figures and Tables

**Table 1:** Primer sequences and accession number

Gene name	Accession number	Primer sequence	Application	TA (°C)	Size of PCR (bp)	Restriction enzyme	SNP	Digested fragments length (bp)
*OLFML3*	NC_019458.2	F: 5′-ATG ATG GCT ACC AGA TTG TC-3′ R: 5′-CTC CTT CTG TAC TGC AGA CT-3′	Genotyping	59	498	*Msp*I (5′-CCGG-3′)	g.90317673 C>T	CC: 195, 303 CT: 195, 303, and 498 TT: 498
*OLFML3*	XM_004002351	F: 5′-TCC AGA GTA GTG AGA GAG AC-3′ R: 5′-ACA AAA GGA ACA AGA TCA GC-3′	qRT-PCR	53	182			
*GAPDH*	NC_019460.2	F: 5′-GAG AAA CCT GCC AAG TAT GA-3′ R: 5′-TAC CAG GAA ATG AGC TTG AC-3′	qRT-PCR	60	203			
*β-Actin*	NC_019471.2	F: 5′GAA AAC GAG ATG AGA TTG GC-3′ F: 5′CCA TCA TAG AGT GGA GTT CG-3′	qRT-PCR	60	194			

TA, annealing temperature; qRT-PCR, quantitative real-time polymerase chain reaction; SNP, single nucleotide polymorphisms; *OLFML3*, olfactomedin like 3; *GAPDH*, glyceraldehyde-3-phosphate dehydrogenase.

**Table 2:** The number of animals per genotype and allele frequency in the *OLFML3* gene of each sheep breed

Sheep breed	N	Genotype frequency			Allele frequency		Chi square (χ^2^)
	
		CC (n)	CT (n)	TT (n)	C	T	
JFT	20	0.45 (9)	0.50 (10)	0.05 (1)	0.70	0.30	0.726
JTT	76	0.36 (27)	0.47 (36)	0.17 (13)	0.59	0.41	0.028
GCS	45	0.09 (4)	0.33 (15)	0.58 (26)	0.26	0.74	0.691
BCS	36	0.61 (22)	0.39 (14)	0.00 (0)	0.81	0.19	2.098
CAS	35	0.77 (27)	0.23 (8)	0.00 (0)	0.89	0.11	0.583
GS	20	0.35 (7)	0.55 (11)	0.10 (2)	0.63	0.37	0.601
JS	96	0.40 (38)	0.47 (45)	0.14 (13)	0.63	0.37	0.003
Total	328	0.41 (134)	0.42 (139)	0.17 (55)	0.62	0.38	3.296

*OLFML3*, olfactomedin like 3; N, number of samples; (..), number of samples which CC, CT, TT genotypes; χ^2^ table, 3.84; JFT, javanese fat tailed; JTT, javanese thin tailed; GCS, garut composite sheep; BCS, barbados cross sheep; CAS, compass agrinak sheep; GS, garut sheep; JS, jonggol sheep.

**Table 3:** Association of the *OLFML3* gene polymorphism with carcass and meat quality in the Indonesian sheep population

Parameters	N	Genotype (μ±SD)		

		CC (n = 29)	CT (n = 53)	TT (n = 18)
Carcass characteristics				
Body weight (kg)	100	24.11±2.91	24.21±3.77	24.27±3.36
Hot carcass weight (kg)	100	9.88±1.90	9.76±2.09	9.47±2.13
Cold carcass weight (kg)	100	8.17±3.69	8.91±2.96	9.28±2.10
Dressing % (kg)	100	42.54±3.93	41.11±5.17	41.30±3.62
Carcass length (cm)	100	63.93±4.72^b^	68.99±10.90^a^	66.66±6.67^ab^
Meat quality	**N**	**CC (n = 28)**	**CT (n = 53)**	**TT (n = 19)**
pH value	100	5.99±0.45	6.12±0.67	5.87±0.55
Tenderness	100	4.29±0.65a	3.75±0.82^b^	3.49±0.64^b^
Cooking loss (%)	100	44.44±8.41^b^	46.44±7.46^ab^	49.42±6.35^a^
WHC (mgH_2_O)	100	85.16±13.92	83.08±7.78	82.88±6.65
WHC (% mgH_2_O)	100	28.38±4.64	27.69±2.59	27.62±2.21

*OLFML3*, olfactomedin like 3; SD, standard deviation; WHC, water holding capacity.

a,b Mean in the same row with different superscripts differ significantly (p<0.05). The Numbers shown in parentheses are the number of individuals with the specified genotype (Duncan’s test).

**Table 4:** Association of *OLFML3* gene polymorphism with the composition of retail meat cut

Variable (g)	Genotype (μ±SE) n = 100		

	CC (n = 40)	CT (n = 43)	TT (n = 17)
Leg	**1,377.30±55.60**	**1,398.50±49.60**	**1,438.00±77.70**
Muscle	917.80±40.20	920.70±38.20	945.80±55.00
Bone	380.60±13.00	391.40±13.40	406.70±19.80
Subcutaneous fats	49.11±6.64	45.27±4.56	51.10±11.90
Intramuscular fats	20.49±1.69	24.60±3.38	21.78±2.67
Pelvic fats	10.83±1.65^b^	12.02±1.87^a^	12.37±2.30^a^
Loin	**315.10±18.30**	**316.20±15.50**	**356.10±26.90**
Muscle	178.60±11.40	186.51±8.78	191.10±15.50
Bone	89.23±6.18	93.23±5.86	113.85±9.03
Subcutaneous fats	21.83±3.96	23.72±3.59	25.28±4.54
Intramuscular fats	5.68±1.33^b^	6.20±1.29^ab^	12.56±4.63^a^
Pelvic fats	19.76±3.59	21.56±3.29	26.65±5.06
Tenderloin	**7.38±1.80**	**2.18±1.07**	**0.00±0.00**
Muscle	7.38±1.80	2.18±1.07	0.00±0.00
Bone	0.00±0.00	0.00±0.00	0.00±0.00
Subcutaneous fats	0.00±0.00	0.00±0.00	0.00±0.00
Intramuscular fats	0.00±0.00^b^	0.00±0.00^b^	0.36±1.50^a^
Pelvic fats	0.00±0.00	0.00±0.00	0.00±0.00
Flank	**91.22±9.76**	**107.53±7.68**	**127.40±10.40**
Muscle	61.76±6.26^b^	79.08±5.89^ab^	92.46±8.46^a^
Bone	0.00±0.00	0.00±0.00	0.10±0.10
Subcutaneous fats	19.71±3.72	18.46±1.91	25.82±3.33
Intramuscular fats:	3.46±0.94	4.70±1.36	4.33±1.11
Pelvic fats	0.00±0.00	0.00±0.00	0.00±0.00
Shoulder	**704.80±39.30**	**732.20±30.70**	**735.70±44.50**
Muscle	459.50±26.60	466.80±19.60	459.10±27.70
Bone	197.70±12.30	189.67±9.68	192.50±19.40
Subcutaneous fats	32.56±4.70	28.56±3.89	22.93±4.68
Intramuscular fats	32.27±4.92	33.72±3.72	36.69±6.51
Pelvic fats	2.00±1.78	0.00±0.00	0.00±0.00
Rack	**331.50±21.20**	**324.80±18.10**	**367.50±28.20**
Muscle	176.80±11.80	189.20±10.50	195.00±18.20
Bone	119.41±6.94	120.79±5.17	130.72±6.97
Subcutaneous fats	18.96±3.93	15.84±2.19	22.90±6.04
Intramuscular fats	8.28±1.23	6.57±1.07	8.59±2.21
Pelvic fats	0.49±0.23	0.05±0.05	0.00±0.00
Breast	**377.10±18.60**	**378.10±16.00**	**387.80±25.30**
Muscle	197.40±10.50	200.73±9.08	195.50±11.90
Bone	121.48±5.30	122.03±5.63	125.36±7.75
Subcutaneous fats	30.89±3.96	27.22±2.54	32.61±6.54
Intramuscular fats	20.79±3.00	22.63±2.38	24.98±4.06
Pelvic fats	0.00±0.00	0.00±0.00	0.00±0.00
Shank	**371.90±15.70**	**358.20±15.60**	**372.90±22.70**
Muscle	206.87±11.20^b^	239.31±9.82^ab^	246.04±15.80^a^
Bone	118.17±4.40	109.82±3.87	112.98±4.99
Subcutaneous fats	10.66±1.54	14.64±1.53	14.72±2.28
Intramuscular fats	7.25±1.04	6.04±0.87	7.29±2.04
Pelvic fats	0.00±0.00	0.00±0.00	0.00±0.00
Neck	**275.50±35.50**	**377.90±26.40**	**411.90±34.40**
Muscle	162.70±21.80	240.70±16.90	238.00±18.80
Bone	81.70±10.60	116.49±9.54	133.80±14.00
Subcutaneous fats	11.07±2.39	19.82±5.74	17.24±2.92
Intramuscular fats	11.48±3.80	8.10±1.46	11.38±1.78
Pelvic fats	0.00±0.00	0.00±0.00	0.00±0.00
Recovery	8,030.00±419.00	8,964.00±461.00	8,594.00±550.00

*OLFML3*, olfactomedin like 3; SE, standard error.

Numbers shown in parentheses are the number of individuals with the specified genotype (Duncan’s test).

a,b Mean in the same row with different superscripts differ significantly (p<0.05).

**Table 5:** Association of *OLFML3* gene polymorphism with the fatty acid composition

Fatty acid composition (%)	Genotype (μ±SD), n = 100		

	CC (n = 44)	CT (n = 39)	TT (n = 17)
Fat content	3.71±3.01	3.84±3.06	3.38±2.51
Caprilic acid (C8:0)	0.03±0.09	0.03±0.08	0.09±0.19
Capric acid (C10:0)	0.09±0.05	0.45±2.23	0.12±0.07
Lauric acid (C12:0)	0.50±0.54	0.41±0.35	0.37±0.33
Tridecanoic acid (C13:0)	0.01±0.01^b^	0.01±0.01^ab^	0.02±0.01^a^
Myristic acid (C14:0)	3.27±1.72	2.93±1.53	2.87±1.20
Myristoleic acid (C14:1)	0.15±0.09	0.14±0.12	0.15±0.11
Pentadecanoic acid (C15:0)	0.51±0.17	0.54±0.18	0.54±0.19
Palmitic acid (C16:0)	18.40±4.79	17.64±5.61	19.26±3.13
Palmitoleic acid (C16:1)	1.35±0.52^a^	1.44±0.54^a^	1.01±0.64^b^
Heptadecanoic acid (C17:0)	0.75±0.28^a^	0.89±0.40^a^	0.55±0.30^b^
Ginkgolic acid (C17:1)	0.25±0.25^a^	0.27±0.35^a^	0.06±0.12^b^
Stearic acid (C18:0)	16.96±5.38	16.87±4.44	18.16±3.27
Elaidic acid (C18:1n9t)	5.16±9.56	2.00±4.42	2.31±1.91
Oleic acid (C18:1n9c)	21.87±11.13	24.67±9.28	25.64±10.92
Linoleic acid (C18:2n6c)	2.53±2.59	2.46±1.07	2.76±0.89
Linolenic acid (C18:3n3)	0.10±0.23^b^	0.11±0.34^b^	0.31±0.46^a^
v-Linolenic acid (C18:3n6)	0.13±0.12	0.12±0.09	0.09±0.08
Arachidic acid (C20:0)	0.05±0.12^b^	0.07±0.12^b^	0.14±0.13^a^
Eicosenoic acid (C20:1)	0.05±0.08^a^	0.02±0.04^ab^	0.02±0.02^b^
Eicosedienoic acid (C20:2)	0.03±0.03	0.04±0.07	0.05±0.04
Cis-8,11,14-Eicosetrienoic acid (C20:3n6)	0.08±0.10	0.06±0.08	0.05±0.03
Arachidonic acid (C20:4n6)	0.01±0.05^b^	0.003±0.010^b^	0.06±0.18^a^
Cis-5,8,11,14,17-Eicosapentaenoic acid (C20:5n3)	0.06±0.11	0.05±0.10	0.01±0.01
Heneicosylic acid (C21:0)	0.40±0.30^ab^	0.35±0.30^b^	0.52±0.28^a^
Behenic acid (C22:0)	0.06±0.07	0.05±0.02	0.05±0.03
Erucic acid (C22:1n9)	0.09±0.14	0.07±0.09	0.06±0.04
Cis-13,16-Docosadienoic acid (C22:2)	0.04±0.06	0.03±0.05	0.02±0.02
Docosahexaaonic acid (C22:6n3)	0.05±0.11	0.04±0.08	0.03±0.03
Tricosanoic (C23:0)	1.04±1.30	1.01±1.54	0.53±0.41
Tetracosanoic acid (C24:0)	0.01±0.05	0.003±0.021	0.005±0.020
Nervonic acid (C24:1)	0.21±0.19^b^	0.24±0.23^b^	0.43±0.32^a^
Fatty acid total	0.06±0.09	0.06±0.05	0.06±0.05
Saturated fatty acid (SFA)	74.79±10.49	73.33±10.74	76.42±14.70
Monounsaturated fatty acid (MUFA)	40.86±7.91	40.08±8.44	42.20±5.08
Polyunsaturated fatty acid (PUFA)	24.52±10.80	27.55±8.36	28.82±10.91
Unsaturated fatty acid (UFA)	4.44±3.42	4.25±2.43	4.43±1.23

*OLFML3*, olfactomedin like 3; SD, standard deviation.

Mean±SD are units of percentage fatty acid composition.

a,b Mean value with different superscript letters in the same row differ significantly at p<0.05.
